# Letter: Sample preparation before sequencing and bioinformatics analysis method selection are more important than the results

**DOI:** 10.1186/s12967-024-05510-2

**Published:** 2024-07-29

**Authors:** Guigeng Tan, Yuanyuan Shao, Zhiguo Zhu

**Affiliations:** Department of Urology, Affiliated Hospital of Jining Medical University, Jining Medical University, Jining, Shandong 272007 China

Dear editor

I read Marianna Talia et al.’s study [1] with great interest. Data sources for the study include RNA-seq data from clinical samples (20 invasive mammary ductal carcinomas and 20 prostate adenocarcinomas) and public datasets (TCGA, METABRIC, AFFYMETRIX, GEO). In this study, authors identified two cancer-associated fibroblasts (CAFs)-related gene signatures for breast and prostate cancer. The CAFs-related gene signatures could serve as an effective survival predictor for patients with breast and prostate cancer. Despite the strengths of this study, several underlying concerns, particularly the experimental design, require further elucidation. We believe that these problems are common but neglected and need to be paid attention to by researchers.

Firstly, the clinical sample’s information needs to be further detailed. This includes not only the clinical pathological characteristics of the patients, but also the way these samples were processed. We found that the authors mixed the 20 breast or prostate cancer CAFs collected into 3 sequencing samples. However, how the technical and biological replicates of the experiment were performed was not disclosed. Unlike the stability of DNA, RNA has strong temporal and spatial properties. Different sample mixing schemes and RNA extraction schemes have a great impact on RNA sequencing results [2]. Figure 1 demonstrated three possible sample processing methods. Obviously, different sample processing methods will inevitably produce different results. Based on our previous experience in extracting CAFs from renal clear cell carcinoma, CAFs extracted from tumor tissues are sufficient for subsequent RNA sequencing. Biological replicates are important in most RNA-seq experiments. Therefore, the act of combining 20 samples into 3 samples greatly weakened the value of the study.

**Fig. 1:**
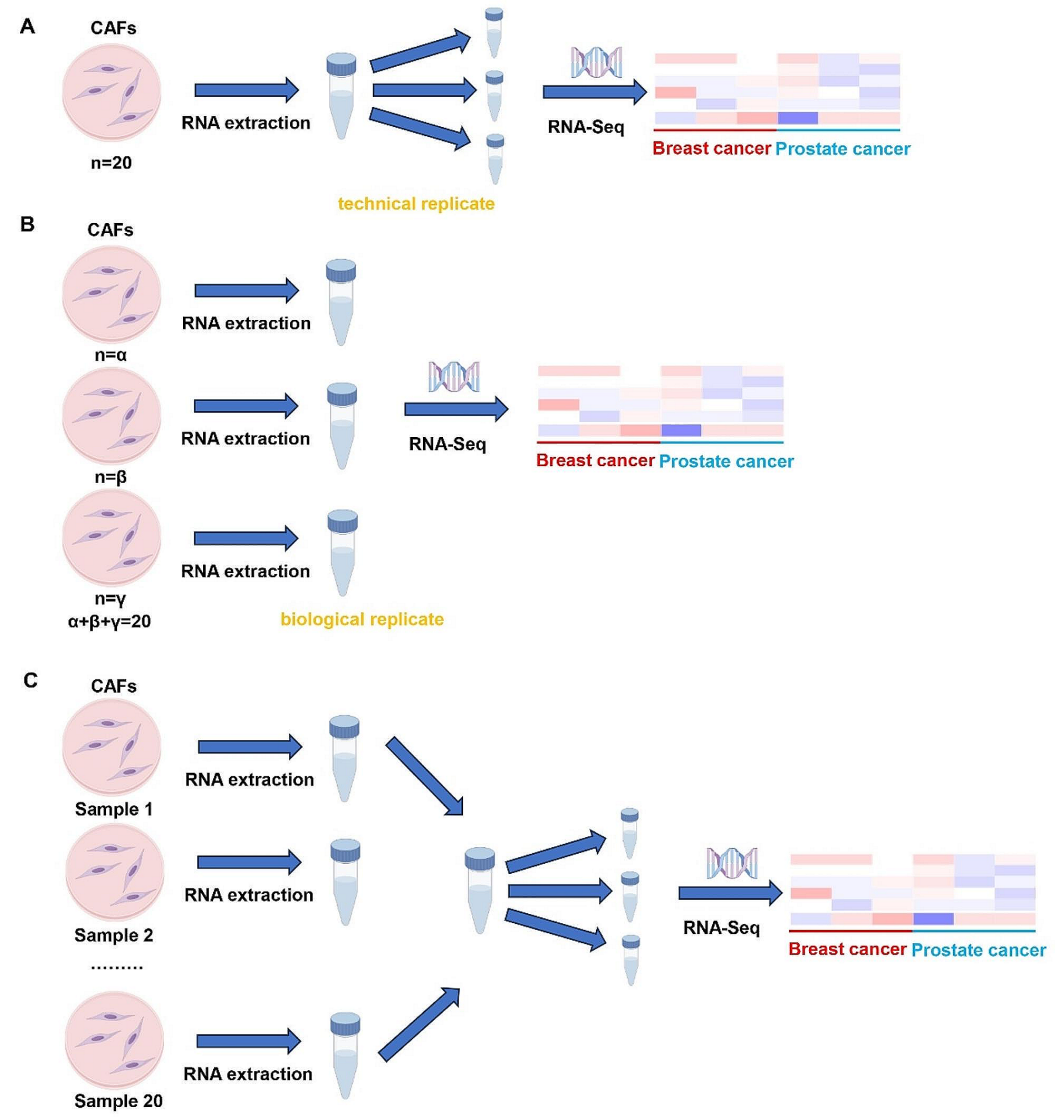
Three possible sample processing methods. **(A)** 20 CAFs samples were mixed and RNA was extracted. The RNA sample divided into 3 sequencing samples The experiment was performed with only technical replicates. **(B)** Considering the long sample collection time, researchers may extract RNA in batches. However, since the number of samples (*n* = 20) is an even number, the amount of CAFs samples contained in each sequencing sample is different. **(C)** RNA was extracted from each CAFs sample separately. The 20 RNA samples were mixed and divided into 3 sequencing samples

Secondly, grouping settings during analysis. We are very confused about why two different CAFs (breast cancer vs. prostate cancer) are compared in the RNA-seq analysis. The article describes that “810 genes were found up-regulated and 1181 genes were found down-regulated in breast with respect to prostate CAFs”. This analysis seems arbitrary and without reason. These differentially expressed genes cannot be considered as tumor-specific CAFs genes. Our analysis should be logical and explainable. In most cases, the differentially expressed genes come from the comparison between CAFs and adjacent normal fibroblasts [3]. ‘FindAllMarkers’ function (for scRNA-seq) and Weighted Gene Co-expression Network Analysis (WGCNA, for RNA-seq) can be used to identify marker genes. In addition, authors intersected genes in CAFs with differentially expressed genes (tumor vs. normal) in TCGA dataset. Using WGCNA to identify key genes originating from CAFs in TCGA dataset seems to be a better choice [4, 5].

Thirdly, the purpose of conducting such research is to obtain a risk model with potential clinical application value. For example, Yan-Jie Zhong et al. [5] establish a CAFs-based risk model: CAFs score = exp (EVA1A) * 0.447 - exp (APBA2) * -0.438 + exp (LRRTM4) *0.284 - exp (GOLGA8M) *1.183 + exp (BPIFB2) *0.870. This risk model could serve as an effective survival predictor for patients with intrahepatic cholangiocarcinoma. In this study, the authors simply provided two lists of genes, with which we can differentiate patients into two groups with different prognoses. This gene list is difficult to quantify and therefore difficult to apply in clinical practice. We also cannot further evaluate its predictive value through a time-dependent ROC analysis.

This letter is not intended to provide a critique of an interesting study. We only want to draw attention to the importance of sample preparation before sequencing and bioinformatics analysis method selection.

## Data Availability

Not applicable.
